# Health status, mental health and air quality: evidence from pensioners in Europe

**DOI:** 10.1007/s11356-018-1534-0

**Published:** 2018-03-10

**Authors:** Eleftherios Giovanis, Oznur Ozdamar

**Affiliations:** 1 Department of Economics, Policy and International Business (EPIB), Manchester Metropolitan University, Business School, Manchester, M15 6BH UK; 2 Faculty of Economics, Department of Econometrics, Adnan Menderes University, Kepez Mevkii, Merkez Kampüsü, 09010 Efeler, Aydın Turkey

**Keywords:** Air pollution, Early retirements, Health status, Old age pensions, Structural equation modelling

## Abstract

Environmental quality is an important determinant of individuals’ well-being and one of the main concerns of the governments is the improvement on air quality and the protection of public health. This is especially the case of sensitive demographic groups, such as the old aged people. However, the question this study attempts to answer is how do individuals value the effects on the environment. The study explores the effects of old and early public pension schemes, as well as the impact of air pollution on health status of retired citizens. The empirical analysis relies on detailed micro-level data derived from the Survey of Health, Ageing and Retirement in Europe (SHARE). As proxies for health, we use the general health status and the Eurod mental health indicator. We examine two air pollutants: the sulphur dioxide (SO_2_) and ground-level ozone (O_3_). Next, we calculate the marginal willingness-to-pay (MWTP) which shows how much the people are willing to pay for improvement in air quality. We apply various quantitative techniques and approaches, including the fixed effects ordinary least squares (OLS) and the fixed effects instrumental variables (IV) approach. The last approach is applied to reduce the endogeneity problem coming from possible reverse causality between the air pollution, pensions and the health outcomes. For robustness check, we apply also a structural equation modelling (SEM) which is proper when the outcomes are latent variables. Based on our favoured IV estimates and the health status, we find that the MWTP values for one unit decrease in SO_2_ and O_3_ are respectively €221 and €88 per year. The respective MWTP values using the Eurod measure are €155 and €68. Overall, improvement of health status implies reduction in health expenditures, and in previous literature, ageing has been traditionally considered the most important determinant. However, this study shows that health lifestyle and socio-economic status, such as education and marital status, are more important, and furthermore, air pollution cannot be ignored in the agenda of policy makers.

## Introduction

Air pollution is one of the most important problems around the globe, with significant adverse effects on health and environment. Air pollution contributes to respiratory and heart diseases, lung cancer and brain damage (Lee et al. 2014). It also causes damage to crops, animals, and contributes to the formation of acid rain (Parson 2003). The WHO report on air pollution shows that yearly almost 7 million people die because of exposure to air pollution, doubling previous estimates and presenting air pollution as the world’s largest single environmental health risk (WHO 2014). In 2012, the number of premature deaths attributable to ground-level ozone in EU-28 reached the 16,000, and 72,000 due to nitrogen dioxides (NO_2_) (EEA 2015). This is the main motivation and urgency of this study due to persistency of the air pollution.

About pension schemes, there have been increases in entitlement ages in several countries because of the continued improvements in life expectancy and the fiscal insolvency of the public pension system. There are two main issues about the retirement age. On one hand, there are conceivable fiscal savings, which can be created by delaying retirement, depending also whether retirement has positive effects on health status. On the other hand, these savings might be partly offset by the increased health expenditures which are associated mainly with age and other factors. Regarding air pollution concentrations, which have significant negative effects on people’s health, a key objective of the environmental legislation in Europe is the air quality improvement.

Overall, concerns for the environmental quality and its impact on people’s well-being date back, since the industrial revolution. The conventional measures employed in the earlier literature review, and most characteristically the gross domestic product (GDP), ignore many non-market determinants that may explain the individual welfare and well-being, including environmental quality and air pollution. Alternative manifestations have been developed, such as using people’s individual well-being as a proxy for utility and the consideration of a rich pool of factors that explain the well-being, besides the income (Deaton 2008; Fleurbaey 2009). However, the income, including also the pension explored in this study, are useful to derive the people’s willingness to pay for improvement in air quality, as we describe in more details in the methodology section.

The objective of this study is to examine the effects of old and early age pensions on health status and mental health measured by Eurod, including air pollution and specifically sulphur dioxide (SO_2_) and ground-level ozone (O_3_) and controlling for weather data, individual and demographic characteristics. Air pollution is examined as an additional determinant of possible raising health expenditures, besides age and other factors. Respondents from 10 countries are examined, using panel data from the Survey of Health, Ageing, and Retirement in Europe (SHARE). The estimated coefficient for the environmental good offers two significant options. First, it offers a direct valuation in terms of the subjective self-reported health and depression. Second, the estimated coefficients for the environmental good and income can be used to calculate the implicit willingness-to-pay (WTP) for the environmental good and the trade-off ratios between the environmental good-air quality and the income.

The study may offer valuable insights to policy makers, about how people evaluate their health in terms of air quality and how much are willing to pay for its improvement. Dolan et al. (2008) argue that subjective well-being data can be used in a number of ways by policymakers by three ways: monitoring progress; informing policy design and policy appraisal. For instance, the former French president Nicholas Sarkozy set up the “Stiglitz Commission” to identify the limits of GDP as an indicator of economic performance and social progress and to account for more relevant indicators of social progress and welfare and presenting the statistical information in a proper way (Stiglitz et al. 2009). While informing policy design is rather common, the evaluation of health status using air pollution and income can be useful to monitor the progress of policies, projects and environmental impact assessment laws, and to further evaluate the effectiveness and performance of policies implemented.

Thus, it is important to improve our understanding of the health status determinants, using large-scale studies, how the people evaluate in monetary values the air quality in terms of their health conditions and how air quality can be influenced directly or indirectly by public policy. Examples of environmental policy and legislations include the Directives 1999/30/EC and 2002/3/EC which define and set up the air quality standards, establish threshold values for concentrations of SO_2_, O_3_ and other air pollutants and proposing programmes and policies.^1^ The reasons of using SO_2_ and O_3_ are similar to other studies, and they rely on the following reasons. First, the main source of SO_2_ is the fossil fuel combustion at power plants, opposed to the other pollutants, such as the particulate matter (PM) and nitrogen oxides (NO_X_). Also, O_3_ can be transferred in other areas, as from rural to urban, easily by the wind. Thus, those air pollutants defined as regional rather than local make much more full use of the regional nature of our data set, since the empirical analysis refers on NUTS and not address or post code level (Luechinger 2009, 2010; Ferreira et al. 2013).

Thus, in this study, the people are not asked how they value the environmental conditions, but they are asked about how healthy they are. Then the econometrics analysis is used to identify how their answers are moved with the environmental conditions. More specifically, individuals are not asked to value the environmental good directly, which is the air quality in this case, but to evaluate their general health status and mental health condition. Therefore, this approach is very similar to the Life Satisfaction Evaluation (LSE) approach (Luechinger 2009; Luechinger and Raschky 2009; Ferreira et al.^2013^; Chongvilaivan and Powdthavee 2012; Giovanis 2014). However, there are major drawbacks using this approach which are discussed in more details in the empirical results section.

The originality and importance of the paper lies on three main aspects. It is the first study that uses regional and large-scale data on air pollution concentrations, considering also other spatial controls, such as weather indicators and type of monitoring stations, but also information about individual and household characteristics, to explain and investigate the health status of old aged and retired people in Europe. Second, it employs various techniques for comparison and robustness checks, which may reduce the endogeneity problem. Third, using those techniques, we estimate the monetary values of the people’s willingness to pay for improvement to their health status, through enhancement of air quality. This can serve as a guide for policy design and implementation, which we discuss in the empirical results section.

The paper is organised as follows. Section 2 presents a short literature review. Section 3 describes the methodological framework. In Section 4 the data and the research sample design are provided. In Section 5 the results of estimating several versions of health functions, with air pollution included, are reported. Moreover, the effects of the air pollutants on health status and their monetary values are reported and discussed. In Section 6 the concluding remarks are presented.

## Literature review

Previous studies have found negative effects of air pollution on health. Following Grossman (1970), the study by Gerking and Stanley (1986) can be considered also as one of the first case studies investigating the relationship between air pollution and health outcomes and the estimation of MWTP. Their study takes place in respondents of the St. Louis survey over the period 1977–1980 and their findings show that the MWTP for a 30% reduction in mean ozone concentrations range between $18 and $25. Another important study in the literature is by Chay and Greenstone (2003) who examined the Clean Air Act Amendments (CAAAs) of 1970 and the plausible improvements in air quality and the causal effect of particulate pollution on infant mortality. The period of study was 1971–1972 and the authors found that the infant mortality rate is reduced by 0.5% for 1 % decrease in air pollution and specifically in total suspended particles (TSP). One of the major first studies that used econometric analysis to model factors that determine subjective well-being is by Grossman (1970) followed by Veenhoven (1993) and Easterlin (2003).

In the epidemiology field, Dockery et al. (1993) expanded their analysis to explore several air pollutants as causes of death from cancer and cardiopulmonary diseases. Following the study by Dockery et al. (1993), several studies in the literature have been carried out to examine the air pollution and its adverse effects on physiological functions, and clinical diseases such as bronchitis, stroke, asthma, lung cancer and relevant deaths (Delfino et al. 1998; Wilhelm and Ritz 2003; O’Neill et al. 2004; Giovanis and Ozdamar 2016). A number of studies also confirm the negative impact of traffic-related pollutants on health and specifically respiratory diseases. The studies by Shima et al. (2003) and Ostro et al. (2006) illustrate that people in Japan residing close to main roads, where the heavy and intensive traffic occurs, present more allergies and respiratory symptoms than those living further away. Similar studies support these findings in USA, Netherlands and UK (Oosterlee et al. 1996; Van Vliet et al. 1997; McConnell et al. 2006) stressing out that high traffic intensity causes increases in respiratory symptoms and reduction in lung function of children, especially due to PM. Like Veenhoven (1993), Easterlin (2003) and Smith and Kington (1997), Hambleton et al. (2005) used the same theoretical framework developed by Grossman to examine determinants of health of elderly—aged 65 and older—in Barbados. More specifically, Hambleton et al. (2005) extended the study of Grossman (1970) and added some alternative factors including geriatric depression, past and current nutrition, and the number of children living outside of household among others. Unlike Grossman’s study, Hambleton et al. (2005) found that current disease conditions accounted for 67.2% of the explained variation in health status of elderly.

Lelieveld et al. (2015) explored the link between premature death and several sources of air pollution in urban and rural environments and they determined that outdoor air pollution led to 3.3 million premature deaths worldwide in 2010 and mostly in Asia. Correia et al. (2013) used a data set for 545 US counties consisting of yearly county-specific average PM_2.5_, yearly county-specific life expectancy and several potentially confounding variables including socio-economic characteristics, smoking prevalence and demographic characteristics for the period 2000–2007. Their findings suggest that a decrease of 10 μg/m^3^ in the concentration of particulate matter PM_2.5_ increases the average life expectancy by 0.35 years. Li et al. (2015) found that while outdoor activity may improve health, the risk of health problems, such as asthma, heart and lung pathologies, is significantly increased when exercising takes place in areas with high air pollution levels. Buck et al. (2013) explored the pollution persistency and the couple fecundity using a cohort of 501 couples. The couples who discontinued contraception to become pregnant was prospectively followed for 12 months of trying to conceive or until pregnancy would be confirmed by a human chorionic gonadotrophin (hCG) test. Their study suggests that a subset of persistent environmental chemicals were associated with reduced fecundity.

Other studies explored the relationship between health status and mental well-being. Hutchinson et al. (2004) examined the social and health factors of 2580 Jamaicans. The life satisfaction and psychological well-being measures have been used in order to proxy their well-being. Bourne (2007, 2008) conducted studies for Jamaican population, considering different groups and sub-groups, examining the determinants of health and well-being. He found that well-being is a function of psychosocial, biological and demographic variables (Bourne 2007, 2008). Despite the contribution of the previous works to the understanding of well-being and the factors of health status, there is a gap in the literature on how the association of air pollution and old and early age pension affect health status.

Previous studies also explored the weather effects on health status and well-being and research is mainly concentrated on heat and cold wave episodes. For instance, the study by Persinger (1980) suggests significant negative correlation between winter temperature and mortality in northern American, northern Asian and European countries. Furthermore, Driscoll (1971) pointed out that high temperature is one of the most important reasons of mortality during summer. Many other studies followed, and support also the association between mortality, health outcomes and temperature (Ellis et al. 1975; Braga et al. 2002; Analitis et al. 2008; Deschenes and Greenstone 2011; Ozdamar and Giovanis 2016).

The most related study to the current one is by Ferreira et al. (2013) who examined the relationship between air pollution and life satisfaction using three waves of the European Social Survey, SO_2_ concentrations and weather conditions. Ferreira et al. (2013) found a negative and significant impact of SO_2_ on life satisfaction. However, this study differs by examining the air pollution effects on general health status and mental health instead of life satisfaction. The study is based on Europe, similarly to the study by Ferreira et al. (2013); nevertheless it adds to the previous literature by using panel data and not cross-sectional. Moreover, the non-movers sample is considered, while the location of interests is based on Nomenclature of Territorial Units for Statistics of level 3 (NUTS 3) and not NUTS 1 or NUTS 2. In addition, additional econometric approaches, such as the ordered Logit random effects and the Blow Up and Cluster (BUC) estimator, are considered for robustness checks. Furthermore, instrumental variables (IV) approach using the Two-Stage Least Squares (2SLS) and the Three-Stage Least Squares (3SLS) methods for air pollution and pension are considered, which have been neglected in previous studies. Moreover, a structural equation modelling (SEM) framework is presented in order to account for the measurement error of the Eurod and to test the causal assumptions of the model.

## Methodology

### Fixed effects

We estimate the following model of health status (*HS*) for individual *i*, in area *j*, at time *t.*

1 $$ {HS}_{i,j,t}={\beta}_0+{\beta}_1\log \left({y}_{i,t}\right)+{\beta}^{\prime }{\mathrm{z}}_{i,j,t}+\gamma {\mathrm{W}}_{j,t}+{\delta}_1{e}_{j,t}+{\mu}_i+{l}_j+{\theta}_t+{l}_jT+{\varepsilon}_{i,j,t} $$

Variable *log(y*_*i,t*_*)* denotes the logarithm of the old age or early age retirement pension, z is a vector of household and demographic factors, discussed in the data section and W is a vector of meteorological variables in location-city in time *t*. The vector *e*_*j,t*_ is the measured emissions of the air pollutant in location *j* and in time *t*. Set *μ*_*i*_ denotes the individual-fixed effects, *l*_*j*_ is the location of NUTS 3 fixed effects, with the exception of Germany which is based on NUTS 2. *θ*_*t*_ is a time-specific vector, while *l*_*j*_ *T* is a set of area-specific time trends which controls for unobservable, time-varying characteristics in the area. Finally, *ε*_*i,j,t*_ expresses the error term which we assume to be *iid*. Standard errors are clustered at the area-specific time trends level.

In its current form, relation (1) can be estimated with ordered Probit or Logit, but only with random effects. In order to estimate fixed effects (FE) models, we apply the Probit-adapted FE technique introduced by van Praag and Ferrer-i-Carbonell (2004). More specifically, Probit-Adapted FE model converts the original ordinal rating of a variable to a continuous distributed variable, and it is calculated based on the frequencies of the ordinal ratings in the sample. In addition, the ordered Logit model with random effects is estimated for robustness check. Another estimator is the FCF developed by Ferrer*-i-*Carbonell and Frijters (2004). However, the “Blow-Up and Cluster” (BUC) estimator is preferred (see Baetschmann et al. 2015 for more technical details^2^) because FCF estimator is inconsistent as the way that by choosing the cutoff point based on the outcome produces a form of endogeneity.

The marginal willingness-to-pay (MWTP) can be derived from differentiating (1) and setting *dHS* = 0. This is the pension-income drop that would lead to the same reduction in health status than an increase in pollution. Thus, the MWTP can be computed as:

2 $$ MWTP=-\frac{dy}{de}=-\frac{\partial f}{\partial e}/\frac{\partial f}{\partial y} $$

Therefore, relation (2) is just the ratio of the partial derivative of health status with respect to air pollution over the partial derivative of health status w.r.t pension-income, which is also defined as the “income equivalent”. This will give us the monetary value of the willingness to pay for a unit decrease in air pollution in order to improve the health outcomes. In other words, employing regression (1) and the MWTP formula (2), it is possible to perform environmental valuation by taking the partial derivative of the health status regression with respect to the environmental variable—air pollution in our case—and placing this effect in terms of the partial derivative of the health status regression with respect to the income-pension variable. Conversely, these estimates can be used to calculate the “compensating income”, that capture the amount of income individuals would be willing to receive following a reduction in air quality. This approach differs from the conventional environmental valuation methods, as the amount derived expresses the experiential preferences of the respondents to the extent that these are captured in the regressions.

In order to understand the technique and the use of the regression (1) and MWTP formula (2), we will use a numerical example by another study. Even though, this example refers to life satisfaction and not health, the procedure is exactly the same, but since there is no study so far exploring the MWTP for health using this method, we present an example using life satisfaction. Ambrey et al. (2014) used the Household Income and Labour Dynamics in Australia survey and pollution data on PM_10_. Their micro-econometric life satisfaction estimates show that the PM_10_ pollutant coefficient is equal at − 0.0159 and 0.1236 for the natural log of household income. Combining these results with the sample mean household income of AUD 40,070, they found that the annual MWTP is equal at AUD 5150. This is the amount that individuals are willing to pay to reduce by 1 day the average number of days, while our estimates express reduction for one unit decreases in the air pollutant. Therefore, the ratio of the air pollutant estimated coefficient (− 0.0159) over the marginal effect of the income estimated coefficient (0.1236) gives a value of 0.1286. Multiplying this ratio by the average income (40,070) gives the estimated willing to pay of AUD 5150.

## Instrumental variables using two-stage least squares and three stage least squares methods

To address the potential endogeneity of retirement, the instruments employed in this study are the same with those applied by other studies (Bloemen et al. 2013; Kapteyn et al. 2013). More specifically, the instrumental variables are two dummy variables indicating whether the respondent is eligible for full or early retirement public pensions using country- and gender-specific pension-eligibility ages as:

3a $$ {Instrument}_{ijt}^{Old\  Age}=1\left( age\ge Statutory\ retirement\  old\ {age}_{jt}\right) $$

3b $$ {Instrument}_{ijt}^{Early\ retirement\  Age}=1\left( age\ge Statutory\ retirement\  old\ {age}_{jt}\right) $$

The dummies, in (3a)-(3b), are equal with one if the individual in location *j* (which belongs in a specific country) and time *t* is equal or older than the statutory retirement age either for old-normal pension or early age pension. Thus, these instruments vary across individuals of different ages in a given country—depending on the individual being above or below the statutory retirement age in the specific country and in a particular year. In order to be valid instruments, the statutory retirement ages must be exogenous and related to retirement decision. Consequently, the statutory retirement age is a governmental policy, as long as public schemes are examined in this study, thus it can be claimed that are exogenous.

Previous studies have shown that these proposed instruments are very strong predictors of retirement behaviour (Rohwedder and Willis 2010; Coe and Zamarro 2011). The instruments used are valid if two conditions are satisfied. First, the instruments have an impact on the probability that individuals receive the treatment, which is the old age and early retirement public pensions. Second, the instruments should not correlate with unobserved factors having an impact on the outcome, which is the self-assessed health status and Eurod. Nevertheless, this instrument exclusion condition might not be testable as Rohwedder and Willis (2010) argue that variations in retirement that are induced by social security incentives are exogenous. Thus, it is important to estimate the causal effects of pension using IV approach. The reason is that confounding problems coming from the correlation between the pension and the error term, resulting from reverse causality or time-varying confounding factors, make difficult to identify the causal effects of pension on health status using conventional FE ordinary least squares (OLS) estimates. Also, in this study, the air pollution is instrumented with the wind direction. The reason is that most probably wind direction affects air pollution but not directly the health status. On the contrary, other weather conditions affect air pollution, but they affect also the health status directly, as we discussed in the literature review section, and these include the temperature, precipitation and wind speed used in this study as possible confounders. The instrumental variable used in this study—wind direction—is influenced by Luechinger (2009), who instrumented for air pollution using the respondent’s location close to large power plants that installed air emissions (SO_2_) control equipment together with wind direction.

Thus, the endogeneity comes for the above-mentioned sorting problems. Even though, the estimates examine the non-movers in order to limit endogeneity which also comes from residential mobility, or by using fixed effects to account for omitted variables an instrumental variable approach is followed. Moreover, as it has been discussed, the spatial level used in this study is NUTS 3, which is not as detailed as post codes are, inducing still the sorting problem. In the case examined, two equations are estimated separately: one for SO_2_ and one for O_3_. Therefore, as the number of equations has to be estimated simultaneously and a problem with endogeneity might be existed, for the reasons mentioned above, a three-stage least square approach will be additionally estimated.

## Structural equation modelling

SEMs with latent variables provide a very general framework for modelling of relationships in multivariate data. SEM is most commonly applied in studies involving latent variables, such as life satisfaction, happiness and health status and they provide a parsimonious framework for covariance structure modelling. In this case, the latent variables examined include the health status and Eurod and SEM is appropriate as these variables are theoretically important, but are not currently measured without substantial measurement error. SEM is an extension of a regression which involves various multiple regression equations that are estimated simultaneously. This provides a more effective and direct way of modelling mediation, indirect effects and other complex relationships among variables and testing for plausible causal assumptions and links among them.

A panel SEM is applied in this study in order to examine whether the proposed causal relationship is consistent with the patterns found among variables in the empirical data. SEM uses a two-step process: the measurement model and the structural equation model. More specifically, the measurement model specifies how the latent (unobserved) variables or hypothetical constructs are measured in terms of the observed variables. Thus, there are two types of variables in SEM: latent and observed variables. Latent variables are variables that are not directly observed or measured, such as hypothetical constructs or factors, including the health status and Eurod used in this study. Happiness, job satisfaction and life satisfaction are other examples of latent variables. They are often inferred from a set of indicators that are directly observed or measured. Therefore, SEM recognises that latent variables are likely measured with error and possibly measured by multiple indicators. So, the measurement equation links the observed variables that serve as indicators to their corresponding latent variables. On the other hand, observed variables are directly measured. Unlike the common linear models, where only observed variables are employed in the analysis, SEM is an analytical procedure which tests how sets of observed variables define latent constructs. The observed variables and unobserved constructs are linked by one- of two-factor equations for observations and in a panel framework it will be for *i* = 1,….., *N* and time *t* = *1*,……, *T*:

4 $$ {x}_{it}={u}_x+{\varLambda}_{\mathrm{x}}{\xi}_{it}+{\delta}_{it}^x $$

5 $$ {y}_{it}={u}_y+{\varLambda}_{\mathrm{y}}{\eta}_{it}+{\delta}_{it}^y $$

Model (4) relates *x*s or *x*_*it*_ = (*x*_*it1*_,……, *x*_*itq*_)΄ to an *n*-vector of latent variables *ξ*_*it*_ = (*ξ*_*it1*_,……, *ξ*_*itn*_)΄, *n ≤ q*, through the *q × n* factor loadings matrix **Λ**_**x**_. Similarly, model (5) relates the vector of indicators *y*_*it*_ = (*y*_*it1*_,……, *y*_*itp*_)΄ to an *m*-vector of latent variables *η*_*it*_ = (*η*_*it1*_,……, *η*_*itm*_)΄, *m ≤ p*, through the *p × m* factor loadings matrix **Λ**_**y**_. The vectors *δ*_*it*_^*x*^ and *δ*_*it*_^*y*^ are the measurement error terms, with dimensions *q ×* 1 and *p ×* 1, respectively, while vectors *u*_*x*_ and *u*_*y*_ are the intercept terms of the models with dimensions *q ×* 1 and *p ×* 1.

Based on the results derived from the measurement models, SEM specifies that causal relationships between the exogenous and endogenous variables and evaluates the amount of unexplained variance among them. On the other hand, the *structural (latent variable)* model is focused on the relationships among latent variables, *n* and *ξ*. This is performed by regressing the dependent vector *n* on the explanatory vector *ξ* as follows for *i* = 1,….., *N* and time *t* = *1*,……, *T*:

6 $$ {\eta}_{it}={a}_i+\mathrm{B}{\eta}_{it}+\varGamma {\xi}_{it}+{\xi}_{it} $$

In that case, *m × m* matrix B describes the relationships among latent variables in *n* and the elements of the diagonal of B are all zero. The *m × n* matrix Γ quantifies the influence of *ξ*_*i*_ on *η*_*i*_, while the *m × 1* vector *ζ*_*it*_ represents the errors in the equation system, while the intercept *a*_*i*_ is the individual-fixed effect. In other words, B is a coefficient matrix relating the endogenous variables to each other, while Γ is a coefficient matrix relating the exogenous to endogenous variables. The common assumptions of model (6) are as follows: the elements of *ξ*_*it*_ and *ζ*_*it*_ are independent and normally distributed, the measurement error vectors *δ*_*it*_^*x*^ and *δ*_*it*_^*y*^ with *δ*_*it*_^*x*^ ∼ *N*_*p*_(0, Σ_x_), Σ_x_ = *diag*(*σ*^*2*^_*1x*_,….. *σ*^*2*^_*qx*_) and *δ*_*i*_^*y*^ ∼ *N*_*q*_ (0, Σ_y_), Σ_y_ = *diag*(*σ*^*2*^_*1y*_,….. *σ*^*2*^_*py*_) are independent. Finally, it is assumed that *Cov*(*ζ*,*δ΄*) = 0, *Cov*(*ξ*,*δ΄*) = 0 and *Cov*(*ξ*,*ζ΄*) = 0, where *δ΄*(*δ*^*x*^*΄*, *δ*^*y*^*΄*).

However, two things should be noticed. First, SEM is a framework that takes two inputs; causal assumptions and empirical data and then derives logical consequences of these inputs—quantitative causal assumptions and statistical measures of fit for testing these assumptions. Thus, causal path analysis and SEM do not necessarily imply causality, which is a main misunderstanding in social sciences (Pearl 2012; Bollen and Pearl 2013). In the case of failure to fit the data well, there is doubt on the strong causal assumptions of zero coefficients. On the other hand, fitting the data well—based on the tests described below—does not “prove” or does not imply the causal assumption (as there is no mathematical proof), but it makes them certainly more plausible. Second, SEM is not equivalent with regression. More specifically, each equation represents a causal link rather than a mere empirical association. On the other hand, in a regression model each equation represents the conditional mean of a dependent variable as a function of explanatory variables (Pearl 2012; Bollen and Pearl 2013).

Next the criteria for model fit assessment are discussed. The first test is the chi square statistic, where the null hypothesis that the model is correct and it has perfect fit in the population is tested. In addition, the evaluation of the model is based on four goodness-of-fit indices: comparative fit index (CFI) developed by Bentler (1990), Tucker-Lewis index (TLI) proposed by Tucker and Lewis (1973), the root mean square error of approximation (RMSEA), and the standardised root mean square residual (SRMR), which is a measure of the mean absolute value of the covariance residuals. The CFI and TLI indices range between 0 and 1 and the larger they are the better the fit is. According to Bentler (1990) and Hu and Bentler (1999), a CFI and TLI value of greater than 0.90 can be expected for a very good fit to the data. Regarding RMSEA, as a rule of thumb, if its value is lower than 0.05 indicates a good fit, values between 0.05 and 0.08 suggest acceptable fit, while values higher than 0.10 imply poor model fit. Values for SRMR less than 0.1 indicate favourable estimates.

**Fig. 1:**
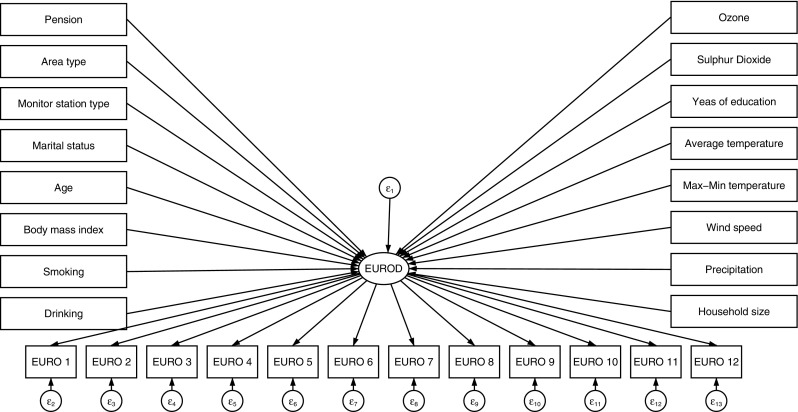
Path diagram and SEM analysis for determinants of Eurod

In Fig. 1, a theoretical SEM model is presented. In this case, the Eurod is the latent dependent variable and the theoretical model examines the health-related quality of life (HRQoL) represented from Eurod. The measurement equation for Eurod consists of 12 symptom domains including depression, pessimism, suicidality among others as it is discussed in the data section. These are represented in Fig. 1 by the variable labels euro1-euro12. The explanatory variables used in the previous regressions are presented and a causal link between them and Eurod is assumed. The health status is not examined as there are no variables to be consisted in a measurement equation. Moreover, the activities of daily living (ADL) and the instrumental activities of daily living (IADL) can be included in the model as additional independent variable and its measurement equation will include variables such as bathing, dressing, mobility, cooking and others. Nevertheless, it is not examined here as the purpose of SEM is to check for robustness of the results derived with the models discussed above.

Thus, SEM allows us to examine and test the causal links between the explanatory variables and Eurod. Secondly, it allows us to include an additional latent variable as explanatory variable to Eurod. Finally, with SEM it is possible to test the validity and reliability of the latent variables examined, Eurod, based on the criteria described above.

Overall, our aim is to implement a set of various econometric methods for sensitivity analysis that will allow us to compare the estimates and to check for robustness. As we mentioned, the purpose of using fixed effects with OLS and employing panel data allow us to identify the model from changes in the pollution level within individuals rather than between individuals. Thus, one advantage of the analysis followed in this study is the panel structure of the data used. In this case, the possible endogeneity bias, due to omitted variable bias and heterogeneity, and which occurs due to the unobservable characteristics of the area that may be correlated with pollution and health status, is eliminated. Furthermore, as we mentioned before, we limit the analysis to the non-movers sample, and this actually gives us the option to control for unobservable characteristics of the area that may be correlated with health and pollution that are fixed over time. The assumption is that the variation in pollution level between interviews is possibly exogenous and driven by differences in the time of the year that the interviews are conducted, which is very logical. Also, the air pollution may be driven by variations in economic activity and weather conditions. However, the “sorting: problem is not totally eliminated with FE-OLS methods, and for this reason we apply IV approach using the 2SLS and 3SLS methods, as well as the SEM approach. This rich set of methods gives us the chance to compare our findings and choose the most favoured ones.

## Data

SHARE is a multidisciplinary and cross-national panel database of micro data on health, socio-economic status and social and family networks of more than 85,000 individuals from 19 European countries aged 50 or over. The principal health outcome is self-assessed health and the possible replies are “excellent/very good/good/fair/poor”. The second principle outcome is the Eurod depression scale with a minimum score of 0 (not depressed) and a maximum of 12 (very depressed). Thus, regarding both outcomes, a negative sign will imply a positive effect of the specific determinant, and vice versa. In order to make the interpretations more convenient, the order of the outcome variables is reversed; health status becomes 1 (poor) to 5 (excellent). Similar, for the Eurod from 1 (very depressed) to 12 (not depressed).

The EURO-D was originally developed to compare symptoms of depression in 11 European centres (Prince et al. 1999). Its items are derived from the Geriatric Mental State examination and cover 12 symptom domains including: depression, pessimism, suicidality, guilty, sleep, irritability, fatigue, appetite, interests, enjoyment, concentration and tearfulness. The psychometric properties of the EURO-D have been extensively investigated and it is found to be reliable and was validated in the study by Guerra et al. (2015) using surveys in nine countries—Cuba, Dominican Republic, Puerto Rico, Peru, Mexico, Venezuela, China, India and Nigeria.

SHARE collects conceptually comparable data in the key domains of demographics, health, work and retirement, income and assets, family and social networks. Currently, at the time of the study, five waves are available (2004/2005, 2006/2007, 2008/2009, 2010–2011 and 2012–2013). However, wave 3 (2008/2009) is a retrospective life history survey with different content than the other three waves and it does not contain the necessary information for the empirical work. Therefore, we use waves 1 (2004–2005), 2 (2006–2007), 4 (2010) and 5 (2012–2013) in our analysis. The first wave of SHARE included 11 European countries (Austria, Belgium, Denmark, France, Germany, Greece, Italy, Netherlands, Spain, Sweden and Switzerland). The second wave added the Czech Republic, Poland and Ireland. The fourth wave added Israel, Estonia, Hungary, Portugal and Slovenia, but Greece abandoned the survey. The countries in the fifth wave are Austria, Belgium, Switzerland, Czech Republic, Germany, Denmark, Estonia, Spain, France, Israel, Italy, Luxembourg, Netherlands, Sweden and Slovenia. Our analysis focuses, however, on the 10 original countries for which we have longitudinal data including waves 1–2, 4 and 5 and these are Austria, Belgium, Denmark, France, Germany, Italy, Netherlands, Spain, Sweden and Switzerland.

The regressions include various personal, household and lifestyle factors, such as pensions, age, marital status, years of education, household size, body mass index (BMI), frequency of the weekdays of alcohol consumption, whether the respondent is a smoker or not, and the type of the area location—whether it is urban, suburban or rural. In addition, weather factors are included into the regression models, such as average, minimum and maximum temperature, precipitation and wind speed. Regarding the air pollution, the SO_2_ and O_3_ are examined as it has been discussed previously. Moreover, the type of the air monitoring station—whether it is background, industrial or traffic—is further included into the regression analysis.

The monthly weather data have been derived by the European Climate Assessment and Dataset and the National Climatic Data Center (NCDC). The air pollution data have been collected by AirBase, the public air quality database system of the European Environmental Agency. The location of air monitoring stations is expressed as point and specifically coordinates. One issue is that the concentrations among the monitoring stations remain unknown due to their uneven distribution. Thus, in order to solve this, the air pollution mapping is based on the inverse distance weighting (IDW), a GIS-based interpolation method. In IDW, the weight of a sampled data point is inversely proportional to its distance from the estimated value. The final level of regional aggregation in the analysis is based on NUTS 3 and NUTS 1 for Germany. The values to vector grids of 10 × 10 km resolution are obtained. The weight function varies from a value of unity at the scatter point to a value approaching zero as the distance from the scatter point increases. The weight functions are normalised so that the weights sum to unity (Franke and Nielson 1980). Two hundred seventy-nine regions and provinces corresponding to 10 countries are used in the analysis. All the monetary values are expressed in Euros with the exception of Denmark, Sweden and Switzerland; thus the pensions have been converted in Euros and deflated in 2013 prices.

Table 1 shows the summary statistics for pension, the health measures and the air pollutants. Overall, the average Eurod mental health of the sample examined is high with average 9.57. The levels are higher especially in Denmark (10.179) followed by Netherlands (10.098) and Sweden (10.047), while the lowest levels are reported in Spain (9.111) followed by France (9.167) and Italy (9.178). On the other hand, the average value of health status is 2.85, with minimum average values again in Spain (2.541), followed by Italy (2.701) and Germany (2.736). The higher average values are presented again in Denmark (3.380) followed by Switzerland (3.330) and Sweden (3.261). In Table 2 the correlation matrix between air pollutants, pensions and well-being measures examined in this study are reported. As it was expected, a higher pension level is associated with higher levels of health status and better mental health—expressed by Eurod—level. Similarly a positive and significant association between Eurod and health status is reported and a similar association is observed for the air pollutants SO_2_ and O_3_. In addition, there is a negative relationship between the air pollutants and the health measures. Finally, a higher level of pension is associated with lower SO_2_ concentrations. This might be explained by the fact that higher income classes are located in areas with cleaner air.

**Table 1:** Summary statistics

Variables	Mean	Standard deviation	Minimum	Maximum
Pension	14,412.05	26,717	212	583,468
Health status	2.842	1.090	1	5
Eurod	9.574	2.280	0	12
SO_2_	5.553	4.937	0.06	103.85
O_3_	50.591	13.536	0.957	117.16

**Table 2:** Correlation matrix for air pollutants, pensions, health status and Eurod measures

	Pension	Health status	Eurod	SO_2_
Health status	0.0169*** (0.000)			
Eurod	0.0295*** (0.000)	0.4227*** (0.000)		
SO_2_	− 0.0126*** (0.000)	− 0.0363*** (0.000)	− 0.0206*** (0.000)	
O_3_	− 0.0075 (0.1556)	− 0.0017** (0.0037)	− 0.0130** (0.0024)	0.0045** (0.0015)

*p* values in brackets, *** and ** denote significant at 1 and 5% significance level

## Empirical results

In Table 3 the estimated results for the model (1) using adapted Probit FE (van Praag and Ferrer-i-Carbonell 2004) are reported. In the first three columns, health status is the dependent variable. In the first two columns, each air pollutant is inserted into the regressions alone, while in column (3) both air pollutants are included. The reason why we follow this procedure is to examine if their impact considerably changes and whether it is possible to disentangle their effects. Similarly, the results for Eurod are reported in Table 4. The effects of the air pollutants do not significantly change when we include them both into the regression analysis. It becomes obvious that their effects on health status are 50–70% higher than their effects on Eurod. This is expected as the last measure refers mainly on mental health, while general health status includes any kind of health problem, including physical and mental.

**Table 3:** Adapted Probit FE for health status

Model	(1) DV: HS	(2) DV: HS	(3) DV: HS
Pension	0.0422*** (0.0085)	0.0421*** (0.0099)	0.0421*** (0.0109)
SO_2_	− 0.0089*** (0.0025)		− 0.0088*** (0.0025)
O_3_		− 0.0038*** (0.0009)	− 0.0036*** (0.0009)
Age	− 0.0128*** (0.0012)	− 0.0122*** (0.0015)	− 0.0124*** (0.0015)
Average temperature	0.0080*** (0.0025)	0.0087*** (0.0021)	0.0084*** (0.0022)
Maximum-minimum temperature	0.0025* (0.0013)	0.0028** (0.0013)	0.0029** (0.0014)
Precipitation	0.0200* (0.0112)	0.0187* (0.0101)	0.0189* (0.0107)
Wind speed	− 0.0210*** (0.0060)	− 0.0201*** (0.0068)	− 0.0206*** (0.0076)
Marital status (reference: married)			
Marital status (single)	0.0289 (0.0207)	0.0176 (0.0158)	0.0195 (0.0161)
Marital status (widowed)	− 0.2358*** (0.0418)	− 0.2225*** (0.0383)	− 0.2348*** (0.0367)
Marital status (divorced)	− 0.1244* (0.0640)	− 0.1126* (0.0590)	− 0.1145* (0.0590)
Marital status (separated)	− 0.0345 (0.1090)	− 0.0372 (0.1118)	− 0.0399 (0.1117)
Years of education	0.0209*** (0.0022)	0.0216*** (0.0027)	0.0226*** (0.0027)
BMI	− 0.0052*** (0.0012)	− 0.0054*** (0.0015)	− 0.0052*** (0.0015)
Smoking (no)	0.0580** (0.0237)	0.0564*** (0.0218)	0.0571*** (0.0208)
How often drinking last 3 months (reference: daily)			
How often drinking (5–6 a week)	0.1901*** (0.0333)	0.1864*** (0.0407)	0.1806*** (0.0401)
How often drinking (1–2 a week)	0.3828*** (0.0275)	0.3808*** (0.0342)	0.3706*** (0.0325)
How often drinking (less than once a month)	0.3374*** (0.0524)	0.3224*** (0.0566)	0.3144*** (0.0545)
Household size	0.0344** (0.0143)	0.0357** (0.0155)	0.0377** (0.0177)
Type of air monitoring station (reference: background)			
Type of air monitoring station (industrial)	− 0.0558** (0.0283)	− 0.0552** (0.0263)	− 0.0551** (0.0281)
Type of air monitoring station (traffic)	− 0.0915*** (0.0286)	− 0.1019*** (0.0297)	− 0.1001*** (0.0337)
Type of area (reference: rural)			
Suburban	0.0363 (0.0288)	0.0505 (0.0310)	0.0326 (0.0420)
Urban	− 0.1671*** (0.0290)	− 0.1729*** (0.0302)	− 0.1709*** (0.0315)
No. observations R square	37,076 0.3760	36,762 0.3652	36,762 0.3083
MWTP SO_2_ (for a unit decrease)	€ 315		€ 312
MWTP O_3_ (for a unit decrease)		€ 137	€ 127

Standard errors between brackets, ***, ** and * indicate significance at 1, 5 and 10% level

**Table 4:** Adapted Probit FE Eurod

Model	(4) DV: Eurod	(5) DV: Eurod	(6) DV: Eurod
Pension	0.0315*** (0.0090)	0.317*** (0.0116)	0.0315*** (0.0104)
SO_2_	− 0.0057** (0.0026)		− 0.0052*** (0.0025)
O_3_		− 0.0022** (0.0009)	− 0.0021** (0.0009)
Age	− 0.0039*** (0.0013)	− 0.0035** (0.0016)	− 0.0036** (0.0016)
Average temperature	0.0069** (0.0029)	0.0065** (0.0027)	0.0061** (0.0028)
Maximum-minimum temperature	0.0030** (0.0013)	0.0031** (0.0013)	0.0030** (0.0014)
Precipitation	0.0158 (0.0117)	0.0154 (0.0119)	0.0153 (0.0119)
Wind speed	− 0.0157** (0.0062)	− 0.0164** (0.0071)	− 0.0161** (0.0069)
Marital status (reference: married)			
Marital status (single)	− 0.2012*** (0.0518)	− 0.1917*** (0.0524)	− 0.1920*** (0.0525)
Marital status (widowed)	− 0.2761*** (0.0293)	− 0.2503*** (0.0320)	− 0.2589*** (0.0360)
Marital status (divorced)	− 0.1500*** (0.0405)	− 0.1492*** (0.0457)	0.1483*** (0.0487)
Marital status (separated)	0.1012 (0.0931)	0.0936 (0.1083)	0.1069 (0.1110)
Years of education	0.0191*** (0.0023)	0.0204*** (0.0029)	0.0202*** (0.0028)
BMI	− 0.0053*** (0.0016)	− 0.0058*** (0.0017)	− 0.0059*** (0.0017)
Smoking (no)	0.0399 (0.0248)	0.0197 (0.0325)	0.0211 (0.0325)
How often drinking last 3 months (reference: daily)			
How often drinking (5–6 a week)	0.1886*** (0.0349)	0.2357*** (0.0430)	0.2329*** (0.0431)
How often drinking (1–2 a week)	0.1880*** (0.0287)	0.1635*** (0.0260)	0.1794*** (0.0271)
How often drinking (less than once a month)	0.2148*** (0.0399)	0.2430*** (0.0433)	0.2392** (0.0594)
Household size	0.0361*** (0.0170)	0.0343* (0.0190)	0.0332* (0.0190)
Type of air monitoring station (reference: background)			
Type of air monitoring station (industrial)	− 0.0815*** (0.0297)	− 0.0903*** (0.0339)	− 0.0821*** (0.0360)
Type of air monitoring station (traffic)	− 0.0145 (0.0301)	− 0.0119 (0.0335)	− 0.0128 (0.0356)
Type of area (reference: rural)			
Suburban	− 0.0512 (0.0380)	− 0.0537 (0.0363)	− 0.0460 (0.0338)
Urban	− 0.0159 (0.0283)	− 0.0197 (0.0319)	− 0.01171 (0.0334)
No. observations R square	36,892 0.3422	36,477 0.3410	35,877 0.3412
MWTP SO_2_ (for a unit decrease)	€ 263		€ 242
MWTP O_3_ (for a unit decrease)		€ 99	€ 98

Standard errors between brackets, ***, ** and * indicate significance at 1, 5 and 10% level

Using the example by Ambrey et al. (2014) in the methodology section, we can similarly derive the MWTP values from the estimates in Table 3. For example in the first column, the SO_2_ estimated coefficient is equal at − 0.0089 and the marginal effect is the same, since the air pollution variable is entered in the regression analysis in linear form. On the other hand, the estimated coefficient of the pension is 0.0422, but the marginal effect is almost 0.41, as the pension is expressed in logarithms, as we presented in the regression (1) in the methodology section. Considering the MWTP formula (2), the ratio gives us the value of 0.0217 and then we multiply with the average pension in the non-movers sample which is roughly €14,500, and this gives us the annual MWTP of €315 for one unit decrease at SO_2._ The value is expressed in annual terms, since also our data are recorded in annual basis, regarding both air pollution and pension-income.

Many epidemiological studies have found negative effects of air pollution on health, especially in respiratory and cardiovascular diseases. These effects can be significant even when the air pollution is below the air quality standard levels (Abelsohn and Stieb 2011; Burt et al. 2013; Chen et al. 2015). On the other hand, the significant effects of air pollution on Eurod and mental health can be explained by the noise pollution coming from traffic and urban areas. Even though the noise pollution is not explicitly examined here, it is associated with high traffic volume, which is also strongly associated with high air pollution levels. Previous studies have demonstrated significant associations between air pollution, depression and suicide (Szyszkowicz et al. 2010; Lim et al. 2012; Mehta et al. 2015; Power et al. 2015). Other studies also have found that indoor air pollution coming from second-hand smokers and biomass fuel leads to depression and perceived stress (Hamer et al. 2010; Banerjee et al. 2012). Similarly, Coe and Zamarro (2011) using the first wave of the SHARE dataset found significant positive effects of retirement on health status. However, their study is limited to cross-sectional analysis, without considering air pollution and weather factors and without limiting the endonegeity problem.

The remained coefficients in Tables 3 and 4 have the expected signs. More specifically, age has been found to be linearly significant, while higher polynomial coefficients are insignificant. Thus, age has significant and negative effects on health status and Eurod, which can be explained to increased health problems associated with old aged people. BMI has a negative impact on both health and Eurod, while smoking has significant and negative effects only on general health status. Drinking seems to be an important determinant for both general health status and mental health, where a monotonic relationship between the frequency of drinking and the probability of reporting a lower level of health status is presented. However, smoking and drinking factors can be subject of criticism as are likely endogenous. More specifically, while a direction effect from those factors to health outcomes can be present, depressed people also may smoke and drink more frequently, while people with health problems, expressed by the health status, are more likely to smoke and drink less.

Regarding the socio-economic factors and weather, the effects have the expected signs. Household size has a positive and significant effect. This is consistent with the study by Ferreira et al. (2013), who found a positive effect of household size and negative impact of number of children. On the other hand, Angeles (2010) found positive and large effect of children on life satisfaction. However, this can be problematic, as based on the data these factors are highly correlated and lead to wrong coefficient signs. For this reason, the number of children has been excluded from the regressions.

In Table 5 the estimated results from alternative approaches are reported, such as BUC, Ordered Logit with random effects IV for pension and air pollutants and 3SLS. The results show that the conventional adapted Probit FE estimates in Tables 3 and 4 are probably not robust. More specifically, the MWTP for SO_2_ and O_3_ range between €312 and 315 and €127 and 137, respectively, for a unit decrease on air pollutants. The results derived from BUC are very close to FE and the respective MWTP values are €299 and €115. Similarly, when we instrument for air pollutants, the MWTP are slightly higher and equal at €327 and €156. The same conclusions are derived with 3SLS. This indicates that either instrumenting for air pollution or not, similar results and MWTP values are obtained. However, on the other hand, the MWTP values are significantly lower when we instrument for the pensions. This is due the fact that the causal effects of pension on health status are higher by almost 70%. This leads to lower MWTP and the denominator of relation (2), which is the partial derivative of health status w.r.t. pension, becomes higher, while the nominator remains almost the same leading to lower MWTP by 30–50%. Overall, the favoured estimates are those by IV for pensions; thus the MWTP €88 and €221 respectively for O_3_ and SO_2_, while the respective MWTP values for Eurod are €68 and €155.

**Table 5:** Robustness checks for health status regressions

Model	BUC	Random effects ordered Logit	FE-IV 2SLS for pension	
Panel A: dependent variable health status				
Pension	0.0496*** (0.0159)	0.0489*** (0.0177)	0.0768*** (0.0157)	
SO_2_	− 0.0083*** (0.0024)	− 0.0079** (0.0035)	− 0.0088*** (0.0025)	
O_3_	− 0.0035** (0.0017)	− 0.0032** (0.0015)	− 0.0036*** (0.0009)	
No. observations	34,682	36,762	32,081	
R square			0.2697	
Wald chi square	10,087 [0.000]	15,503.96 [0.000]		
Sargan exogeneity test			0.1122 [0.8112]	
Weak indentification test			15.626 [0.0154]	
MWTP SO_2_ (for a unit decrease)	€ 299	€ 292	€ 221	
MWTP O_3_ (for a unit decrease)	€ 115	€ 108	€ 88	
Panel B: dependent variable Eurod				
Pension	0.0473** (0.0222)	0.0451** (0.0105)	0.0527*** (0.0202)	
SO_2_	− 0.0053*** (0.0015)	− 0.0051** (0.0019)	− 0.0055** (0.0026)	
O_3_	− 0.0017* (0.0010)	− 0.0017* (0.0010)	− 0.0022* (0.0012)	
No. observations	33,785	35,877	30,966	
R square			0.2386	
Wald chi square		13,136.85 [0.000]		
Exogeneity test			0.229 [0.6324]	
Weak instrument test			17.910 [0.0235]	
MWTP SO_2_ (for a unit decrease)	€ 239	€ 244	0.8341 € 155	
MWTP O_3_ (for a unit decrease)	€ 81	€ 84	€ 68	
Model	FE-IV 2SLS for SO_2_	FE-IV 2SLS for O_3_	3SLS SO_2_	3SLS O_3_
Panel A: dependent variable health status				
Pension	0.0418** (0.0196)	0.0419** (0.0189)	0.0419*** (0.0056)	0.0419*** (0.0059)
SO_2_	− 0.0091** (0.0043)		− 0.0092*** (0.0042)	
O_3_		− 0.0044** (0.0019)		− 0.0039*** (0.0017)
No. observations	30,566	30,139	35,877	36,762
R square	0.2511	0.2503	0.2804	0.2713
Wald chi square				
Sargan exogeneity test	0.604 [0.4372]	0.668 [0.4139]		
Weak indentification test	16.389 [0.0129]	16.735 [0.0121]		
MWTP SO_2_ (for a unit decrease)	€ 327		€ 326	
MWTP O_3_ (for a unit decrease)		€ 156		€ 144
Panel B: dependent variable Eurod				
Pension	0.0312** (0.0146)	0.0314** (0.0147)	0.0316*** (0.0051)	0.0319*** (0.0056)
SO_2_	− 0.0061* (0.0032)		− 0.0063** (0.0031)	
O_3_		− 0.0025** (0.0012)		− 0.0029** (0.0012)
No. observations	30,062	29,627	34,923	35,787
R square	0.2354	0.2171	0.2393	0.2251
Wald chi square				
Exogeneity test	2.021 [0.1551]	0.383 [0.5363]		
Weak instrument test	12.007 [0.0423]	12.378 [0.0393]		
MWTP SO_2_ (for a unit decrease)	€ 269		€ 275	
MWTP O_3_ (for a unit decrease)		€ 116		€ 119

Standard errors between brackets, *p* values between square brackets. ***, ** and * indicate significance at 1, 5 and 10% level

In Table 6, the SEM results are reported. The pension effect is close to the one derived by IV for pension. The effects of the remained coefficients remain the same with those found by FE. Concluding the MWTP for O_3_ and SO_2_ are €57 and €145, respectively, very close to the MWTP values found when the pension is instrumented which are equal at €165 and €68 for O_3_ and SO_2,_ respectively. Regarding the goodness of fit of the model, based on CFI and TLI, with values 0.962 and 0.958, which are very close to 1, it is concluded that the model fits the data very well. Also, RMSEA is significantly lower than the proposed value of 0.05 and it is equal at 0.0068, while RMSR of 0.010 is much lower than the proposed rule of thumb of 0.1. Finally, based on the χ^2^/df, where *df* denotes the degree of freedoms is 2.85 and its *p* value is equal at 0.463. Thus, the null hypothesis of the goodness of fit of the model is accepted.

**Table 6:** SEM estimates for Eurod

Variables	Coefficients		Coefficients
Pension	0.0493*** (0.0084)	How often drinking last 3 months (reference: daily)	
SO_2_	− 0.0054** (0.0023)	How often drinking (5–6 a week)	0.2494** (0.1041)
O_3_	− 0.0020*** (0.0005)	How often drinking (1–2 a week)	0.1861** (0.0824)
Age	− 0.0036** (0.0016)	How often drinking (less than once a month)	0.2208 (0.1340)
Average temperature	0.0069** (0.0029)	Household size	0.0233** (0.0100)
Maximum-minimum temperature	0.0033* (0.0017)	Type of air monitoring station (reference: background)	
Precipitation	0.0128 (0.0106)	Type of air monitoring station (industrial)	− 0.0596*** (0.218)
Wind speed	− 0.0136** (0.0062)	Type of air monitoring station (traffic)	− 0.0235 (0.0203)
Marital status (reference: married)		Type of area (reference: rural) Suburban	− 0.0122 (0.0205)
Marital status (single)	− 0.1852** (0.0758)	Urban	− 0.0150 (0.0189)
		No. observations	30,792
Marital status (widowed)	− 0.2214*** (0.0464)	MWTP SO_2_ (for a unit decrease)	€ 145
Marital status (divorced)	0.1190*** (0.0289)	MWTP O_3_ (for a unit decrease)	€ 57
		χ^2^/df	2.85 [0.463]
Marital status (separated)	0.1280 (0.1205)	CFI	0.862
Years of education	0.0281*** (0.0021)	TLI	0.858
		RMSEA	0.0068
BMI	− 0.0098*** (0.0028)	RMSR	0.0100
Smoking (no)	0.1860* (0.1005)		

Standard errors between brackets, *p* value between square brackets. ***, ** and * indicate significance at 1, 5 and 10% level

In the previous literature (e.g. Meijer et al. 2013), it is discussed that ageing is one of the most important factors of health expenditures. However, the results in this study, acknowledging the fact that improvement in health leads to reduction of health expenditures, show that other factors are more important, including health lifestyle, such as drinking, smoking and BMI, socio-economic characteristics, like education and marital status, showing the possible social exclusion of some groups, i.e. low educated, widowed and others. Finally, even though are not more important than the age factor, air pollutants cannot be ignored and relevant policy implementations for air quality improvement are needed.

More specifically, various policy implications are derived from the empirical results so far. Regarding age, we have shown that older people are more likely to report lower levels of health status, which is one of the most important policy concerns for the future fiscal viability and sustainability of the health, pension and insurance systems, both public and private. Furthermore, the demographic transition observed in Europe, such as the increasing number of people working for less than half a lifetime, due to extended period of training and education in early life, early retirement schemes and prolonged life expectance and longevity, poses challenges to the sustainability of health and pension systems. Policy makers should intervene in labour force participation, investment and saving behaviour that may increase the fiscal viability and sustainability. Incentives provided by the government and employers can play a major role in determining the labour force participation. For instance, reducing the hiring costs and the implicit tax on continuing work beyond the normal retirement age and in combination with the improvement on medical technology and treatment will give the potential to older worker to encourage longer working lives. Changes in investment and savings behaviour of people can be crucial as healthy lifestyle promotion, disease prevention programs and health enhancement in early life may prolong the working lives, but also to enhance the financial and wealth situation of old aged people. This is in line with the remained empirical findings. In particular, we have shown that more educated and wealthier people, non-smokers, non-heavy drinkers and non-obese are more likely to report higher levels of health status. This illustrates that more educated people have higher earning potentials and are more aware about the benefits of a healthy lifestyle.

The prevalence of obesity is a major challenge, not just for the public health, but for governance and decision making, as it leads to a high risk of cardiovascular diseases (Kopelman 2007). There are various policy options, including planning procedures, such as the increase in “walkability” and “cyclability” of the built environment and the improvement of safety from the points of view of crime and traffic. Furthermore, targeted interventions, such as when children are young, are very important, as the obesity in adults is more likely to be followed by childhood obesity. Implementations of population-wide interventions improving the health of the population as a whole and focus and information about the health consequences of obesity, rather than the obesity itself, are other policy options. Other choices include the introduction of programmes increasing food literacy and taxes on obesity-promoting foods which policies can be applied also for alcohol and smoking (Butland et al. 2007; Chipperfield et al. 2007).

Also, the empirical results show that people located in urban areas present lower levels of health status. This can be explained by the fact that urban areas are more polluted, due to increased population, traffic and increased motor vehicle traffic, and other related air pollutants, as well as noise pollution that come from traffic- and construction-related activities. Furthermore, urban areas can be associated with sub-standard housing insufficient or contaminated drinking water, inadequate solid waste disposal services industrial waste, and stress associated with poverty and unemployment. Therefore, good and sustainable urban planning and design of smart cities, exchange of best practice models and the determination and leadership of stakeholders across disciplines, sectors, communities, public authorities and countries will be critical elements of success. This includes the design and implementation of policies that reduce air pollution and traffic, such as the congestion changing zones, investments in infrastructure that encourage walking and cycling, teleworking employment schemes that allow the employees to work at home some days of the month, and policies that reduce crime and enhance safety. Improvement on public transit and promotion of walking as healthy lifestyle are other options. Alternative policy measures that are strongly related also with air pollution include the use of hybrid and electric cars and investments on grid supply energy network for them, use of alternative resources of energy and retrofitting homes by incentives leading to energy efficiency improvement.

As we have shown earlier, widowed and divorced report lower levels of health status compared to singles and married. This can be explained by the fact that these individuals are old and also less wealthy, which is associated with loss of income due to the death of the spouse. Furthermore, the loss of the spouse increases the depression and deteriorates the health conditions. In this case, support groups and survivor benefits may enhance the health outcomes. A policy option could be also the promotion of training and education programmes that increase physical activities that reduce the probability occurrence of the partner’s death.

The robust evaluation of preferences and willingness to pay is a very useful tool for valuing intangible goods or environmental quality (Carson 2000; Krupnick et al. 2002; Wang and Mullahy, 2006; Alberini and Chiabai 2007; Mahmud 2009). Therefore, estimates and measurements to valuate losses air pollution are relevant and valuable in policy-making, implementation and in validating environmental policy changes, because of the pollution impacts can human health and the costs of public policies (Jin-Lee et al. 2011).

So far we described the advantages of using various econometric methods and approaches, and briefly we mentioned the merits of the LSE approach. Next we will discuss in more details its advantages and drawbacks. This approach offers advantages over the more conventional environmental valuation techniques, which mainly include the hedonic pricing and contingent valuation methods. As we discussed in the introduction section, LSE and therefore in this case health status do not require awareness of cause-effect, and do not ask the respondent’s evaluation of the environmental good, but only her health status. Then, the econometric estimation allows us to explore how health status is affected by air pollution across time. These changes can be driven by observed or unobserved pollution variation and therefore this approach is closely related to hedonic property pricing, but it relies on health status rather than house price to evaluate how individuals value their environment, and thus requires less stringent assumptions. Also, it does not rely on the ability of the respondents to account and consider all the relevant consequences of a change in the provision of a public good. Obviously, health outcomes may be correlated with some unobserved amenities that also affect pollution level, and in cross-section, the estimates may thus be biased. Instead, we rely on individual level panel data, so that unobserved individual level and geographical characteristics can be accounted for. The identification then comes from variation in pollution level between interviews as we described in the methodology section.

Regarding the hedonic pricing, the weak results may be explained by two econometric identification problems. First, it is likely that the estimated association between housing price and air pollution is biased due to omitted variables. Second, if there is heterogeneity across individuals in preferences for clean air, then individuals may self-select into locations based on these unobserved differences (Chay and Greenstone 2005) which is also the “sorting” issue we discussed earlier and is a concern for the present study. Additionally, this approach is subject to market distortion because MWTP is a crude average of the marginal values estimated under specific circumstances, as it relies on the assumption that the housing market should be on equilibrium (Smith and Huang 1995; Frey et al. 2009). Indeed the housing market may well be correlated with pollution, leading to selection bias in hedonic pricing analysis. Another major advantage of the LSE over the hedonic property pricing method is that mistaken perceptions about the environmental (dis) amenity are avoided, which may otherwise lead to biased estimates and not precise monetary values.

Concerning the second traditional environmental evaluation method, the contingent valuation approach, there are also criticisms. One argument is that individuals do not have adequate understanding of what they are being asked to evaluate, which is not the case in the LSE approach, since the individuals are not directly asked to evaluate the environmental good. Another disadvantage is that the individuals might have limited or poor incentives to disclose their true demand (Luechinger 2009; Frey et al. 2009; MacKerron and Mourato 2009). More specifically, using contingent methods, biases may arise due the lack of real monetary incentives and credible policy mechanisms.

In addition the answers may depend substantially on the form in which questions are posed. Direct questioning or contingent valuation studies ask respondents to value an output, such as a day spent in an activity, rather than a change in pollution concentrations per se (Croper and Oates 1992). Also if the commodity to be valued is not well understood, contingent valuation responses are likely to be unreliable. More specifically, responses tend to exhibit wide variation, and respondents may even prefer less of a good to more, especially when there are open-ended questions for a good which is not traded in conventional markets, as the air quality (Croper and Oates 1992). This is especially the case of environmental goods, as the LSE approach avoids the problem of how to make the environmental issue understandable to the population of interest, which is a task that can be particularly very difficult and complicated when valuing environmental goods such as air and water pollution and even more for specific air pollutants, without knowing or understanding their main effects on health (Christie et al. 2006).

Overall, there are three main issues with the contingent approach. First, the people are not willing to pay for an improvement in air pollution if there is no monetary motivation. Second, they are likely to report lower monetary values to avoid possible high taxes for the collection of funds that would be allocated for improvement in environment. This is associated with strategic behaviour, such as free riding, where an individual anticipates that other people will pay for the air quality improvement and therefore (s)he will enjoy the same benefits of clean air. This will lead to significant biased estimates of the willingness to pay. Third, and most important, people should be convinced that the money paid is really invested to policy implementation for changes in environmental conditions (Kahneman et al. 2006; Welsch and Kuhling 2009).

Another main characteristic and a valuable feature to LSE and the effects of air pollution on health outcomes is the development and advantages of the geographic information systems (GIS) and the availability of spatially referenced data. This has been a very useful input, as it makes possible to link the environmental conditions to individual’s environmental and location in household panel surveys. The air pollution mapping of this type allows accurate modelling and precise estimates (Conley 1999; Tietenberg and Lewis 2009).

However, besides the merits of the LSE approach and the empirical methods employed, there are also drawbacks. The first issue is that using health status and Eurod measures must be comparable across groups of the same individuals under different circumstances. This is due the fact that the self-reported answers are highly heterogeneous among individuals, which is explained by the different scales adopted by them when evaluating their health. This phenomenon is known as Differential Item Functioning (DIF) bias. One possible solution is to employ anchoring vignettes, which are available in SHARE dataset; however, they cover only the first wave. The purpose of this study is to use panel data in order to examine the effects of air pollution on health dynamically across a period of time and not statically using only one wave. Thus, this research relies on inter-temporal comparisons of health within individuals and it is assumed that the scale and the interpretation of the question by a respondent remains the same between survey waves, which reduces the potential bias associated with DIF. This is the reason why we implement the fixed effects model. In addition, the DIF can be detected through the SEM proposed in this study, including self-reported variables, such as health status, and life satisfaction among others that are characterised by measurement error. However, based on the available data, SEM analysis takes place only for the Eurod and not for health status.

Second, another limitation is the fact that people may choose where they reside. One way to limit this sorting problem and the possible endogeneity derived from the “sorting” issue is to consider only non-movers. This would be efficient if the exact location of the respondent is known, such as post code or address. However, the highest spatially level used in this study is NUTS 3. Thus, the population of interest is limited to non-movers in order to limit endogeneity. Another possible way to reduce the endogeneity is to apply 2SLS and 3SLS methods using wind direction as an instrument for air pollution, as it is discussed in the methodology section. Nevertheless, the issue cannot be totally eliminated, mainly because of two main reasons. First, it is unknown whether the respondents are located in areas with clean air because are richer, or more or less risk averted to air pollution due to health causes. One solution could be to select only the respondents that are moving from more polluted to cleaner areas, and vice versa, and to investigate the causes of the relocation and whether air pollution is one of those. However, this will considerably limit the sample at a large degree, restricting our interest also to the remained determinants of health outcomes. On the other hand, considering only the non-movers sample, we do not have information about their relocation status, before the SHARE project started. Second, is the low disaggregation geographical area level which is NUTS 2, while a highly disaggregated geographical level, such as post code level or neighbourhood would provide much more detailed air pollution mapping and therefore, much more precise estimates. Concerning therefore, the air pollution mapping, but also the time frame which is based on yearly basis, this study presents the same issues with the study by Luechinger (2009) and Ferreira et al. (2013). On the contrary, the studies by Giovanis (2014) and Giovanis and Ozdamar (2016) are based on municipality zip codes in Switzerland and local authority districts in UK.

Another important issue is that pensions, similar to income, may have a reverse causal effect on health status and mental health. Pischke (2011) finds evidence of causal effects of income on life satisfaction; however, he argues that also happier people might earn more. Similarly, in this case examined, a possible degree of reverse causality between pension and health might exist. On the one hand, it could be argued that pensions are fixed. However, a healthy person might be willing to work additional years, resulting in higher pension levels. Furthermore, healthier people can be more productive having higher earning potentials, and thus, leading to higher pension-income. On the other hand, a person who is already mentally ill or reports lower level of health status might be willing to get retired early for various reasons. Therefore, to reduce this source of endogeneity bias, we applied IV and SEM approaches.

## Conclusions

This study has attempted to contribute in the literature by examining the effects of pensions and air pollution on health status. Moreover it aimed to fill the gap in the literature regarding the heterogeneous effects of pensions and air pollution, as well as of additional socio-economic determinants and weather conditions on health. Two main important points are revealed. First, air pollution has direct and significant effects on individuals’ health status Second, there is evidence of a substantial trade-off between pension and air quality, which is the compensating differential for air pollution.

However, this study is not without limitations. First and most important issue is the air pollution assignment and mapping regarding the location and time frame. More specifically, the air pollutants are examined in annual concentration levels, such as in the case of Ferreira et al. (2013) and Luechinger (2009). A higher frequency such as daily, weekly or monthly, makes air pollution assignment more exogenous. The geographical mapping of the air pollution is another issue. A higher spatially level, such as post codes, could lead to far more precise estimates of the air pollutant coefficients. Even this is not feasible in European level, it is suggested for future research. Lastly, knowing the effects of air pollution on health estimates on hospitalisation can take place. Then based on these estimates, the hospitalisation costs caused by air pollution can be estimated. Thus, knowing the MWTP and the hospitalisation costs, it would be possible to estimate a cost-benefit analysis. This is possible using SHARE; however, it is out of this study’s scope. Moreover, estimating the air pollution reduction costs, i.e. congestion zones reducing traffic, air pollution prevention controls for industry are very difficult to be estimated and to be known at European level. Therefore, using high spatially disaggregated data will help research and policy makers to clarify the potentially complex links between health and individuals’ exposure to air pollution. This could offer further insights for achieving simultaneously healthier, cleaner and more sustainable cities.

## Footnotes

1
http://ec.europa.eu/environment/air/quality

2 This method has been developed by Baetschmann et al. (2015) and it is described in much more details in their paper

## References

[CR1] Abelsohn A, Stieb DM (2011). Health effects of outdoor air pollution. Approach to counseling patients using the Air Quality Health Index. Can Fam Physician.

[CR2] Alberini A, Chiabai A (2007). Urban environmental health and sensitive populations: how much are the Italians willing to pay to reduce their risk. Reg Sci Urban Econ.

[CR3] Ambrey C, Fleming C, Chan A (2014). Estimating the cost of air pollution in south East Queensland: an application of the life satisfaction non-market valuation approach. Ecol Econ.

[CR4] Analitis A, Katsouyanni K, Biggeri A, Baccini M, Forsberg B, Bisanti L, Kirchmayer U, Ballester F, Cadum E, Goodman PG, Hojs A, Sunyer J, Tiittanen P, Michelozzi P (2008). Effects of cold weather on mortality: results from 15 European cities within the PHEWE project. Am J Epidemiol.

[CR5] Angeles L (2010). Children and life satisfaction. J Happiness Stud.

[CR6] Baetschmann G, Staub EK, Winkelmann R (2015). Consistent estimation of the fixed effects ordered logit model. J R Stat Soc Ser A.

[CR7] Banerjee M, Siddique S, Dutta A, Mukherjee B, Ranjan M (2012). Cooking with biomass increases the risk of depression in pre-menopausal women in India. Soc Sci Med.

[CR8] Bentler PM (1990). Comparative fit indexes in structural models. Psychol Bull.

[CR9] Bloemen H, Hochguertel S, Zweerink J (2013) The causal effect of retirement on mortality: evidence from targeted incentives to retire early IZA DP No 7570 10.1002/hec.3493 28233361

[CR10] Bollen KA, Pearl J (2013) Eight Myths About Causality and Structural Equation Models. In: SL Morgan (ed) Handbook of Causal Analysis for Social Research, Chapter 15. Berlin, Springer, pp 301–Q12328

[CR11] Bourne P (2007). Using the biopsychosocial model to evaluate the wellbeing of the Jamaican elderly. West Indian Med J.

[CR12] Bourne P (2008). Health determinants: using secondary data to model predictors of well-being of Jamaicans. West Indian Med J.

[CR13] Braga ALF, Zanobetti A, Schwartz J (2002). The effect of weather on respiratory and cardiovascular deaths in 12 U.S. cities. Environ Health Perspect.

[CR14] Buck LGM, Sundaram R, Schisterman EF, Sweeney AM, Lynch CD, Gore-Langton RE, Maisog J, Kim S, Chen Z, Barr DB (2013). Persistent environmental pollutants and couple fecundity. Environ Health Perspect.

[CR15] Scientific Evidence of Health Effects from Coal Use in Energy Generation. Healthcare Research Collaborative. University of Illinois at Chicago School of Public Health Chicago, Illinois

[CR16] Butland B, Jebb S, Kopelman P, McPherson K, Thomas S, Mardell J, Parry V (2007). Foresight tackling obesities: future choices—project report.

[CR17] Carson R (2000). Contingent valuation: a user’s guide. Environ Sci Technol.

[CR18] Chay K, Greenstone M (2003) Air quality, infant mortality, and the Clean Air Act of 1970, NBER Working Paper No. 10053, Cambridge, MA

[CR19] Chay KY, Greenstone M (2005). Does air quality matter? Evidence from the housing market. J Polit Econ.

[CR20] Chen R, Kan H, Chen B, Huang W, Bai Z, Song G, Pan G (2015). Association of particulate air pollution with daily mortality. The China air pollution and health effects study. Am J Epidemiol.

[CR21] Chipperfield T, O’Brien R, Bolderson T, Eidinow E, Shafner L, Butland B (2007). Qualitative modelling of policy options foresight tackling obesities: future choices.

[CR22] Chongvilaivan A, Powdthavee N (2012). Do different work characteristics have different distributional impacts on job satisfaction? A study of slope heterogeneity in workers. Well-being. Br J Ind Relat.

[CR23] Christie M, Hanley N, Warren J, Murphy K, Wright R, Hyde T (2006). Valuing the diversity of biodiversity. Ecological Economics. 58(2):304–317

[CR24] Coe B, Zamarro G (2011). Retirement effects on health in Europe. J Health Econ.

[CR25] Conley T (1999). GMM estimation with cross sectional dependence. J Econ.

[CR26] Correia WA, Pope AC, Dockery D, Wang Y, Ezzati M, Dominici F (2013). Effect of air pollution control on life expectancy in the United States: an analysis of 545 U.S. counties for the period from 2000 to 2007. Epidemiology.

[CR27] Croper ML, Oates WE (1992). Environmental economics: a survey. J Econ Lit.

[CR28] Deaton A (2008). Income, health, and well-being around the world: evidence from the Gallup World Poll. J Econ Perspect.

[CR29] Delfino RJ, Murphy-Moulton AM, Becklake MR (1998). Emergency room visits for respiratory illnesses among the elderly in Montreal: association with low level ozone exposure. Environ Res Sect A.

[CR30] Deschenes O, Greenstone M (2011). Climate change, mortality, and adaptation: evidence from annual fluctuations in weather in the US. Am Econ J Appl Econ.

[CR31] Dockery D, Pope CA, Xiping X, Spengler J, Ware J, Fay M, Ferris B, Speizer F (1993). An association between air pollution and mortality in six U.S. cities. N Engl J Med.

[CR32] Dolan P, Peasgood T, White M (2008). Do we really know what makes us happy? A review of the economic literature on the factors associated with subjective well-being. J Econ Psychol.

[CR33] Driscoll DM (1971). Base lines for measuring adverse effects of air pollution: some evidence for weather effects on mortality. Environ Res.

[CR34] Easterlin RA (2003) Building a better theory of well-being. Prepared for presentation at the conference Paradoxes of Happiness in Economics, University of Milano-Bicocca, March 21–23

[CR35] EEA (2015) Air quality in Europe—2015 report. EEA Report No 5/2015. European Environmental Agency, Denmark, https://www.eea.europa.eu/publications/air-quality-in-europe-2015/at_download/file

[CR36] Ellis FP, Nelson F, Pincus L (1975). Mortality during heat wave in New York City, July 1972 and August and September 1973. Environ Res.

[CR37] Ferreira S, Akay A, Brereton F, Cuñado J, Martinsson P, Moro M, Ningal TF (2013). Life satisfaction and air quality in Europe. Ecol Econ.

[CR38] Ferrer-i-Carbonell A, Frijters P (2004). How important is methodology for the estimates of the determinants of happiness?. Econ J.

[CR39] Fleurbaey M (2009). Beyond GDP: the quest for a measure of social welfare. J Econ Lit.

[CR40] Franke R, Nielson G (1980). Smooth interpolation of large sets of scattered data. Int J Numer Methods Eng.

[CR41] Frey BS, Luechinger S, Stutzer A (2009) The life satisfaction approach to environmental valuation. IZA Discussion Paper Series No. 4478

[CR42] Gerking S, Stanley RL (1986). An economic analysis of air pollution and health: the case of St. Louis. Rev Econ Stat.

[CR43] Giovanis E (2014). Relationship between well-being and recycling rates: evidence from life satisfaction approach in Britain. J Environ Econ Policy.

[CR44] Giovanis E, Ozdamar O (2016). The impact of air pollution on health problems in Britain. Int J Sustain Econ.

[CR45] Grossman M (1970) The demand for health: a theoretical and empirical investigation. Ph.D. dissertation, Columbia University

[CR46] Guerra M, Ferri C, Llibre J, Prina MA, Prince M (2015). Psychometric properties of EURO-D, a geriatric depression scale: a cross-cultural validation study. BMC Psychiatry.

[CR47] Hambleton IR, Clarke K, Broome HL, Fraser HS, Brathwaite F, Hennis AJ (2005). Historical and current predictors of self-reported health status among elderly persons in Barbados. Rev Panam Salud Publica.

[CR48] Hamer M, Stamatakis E, Batty DG (2010). Objectively assessed second-hand smoke exposure and mental health in adults: cross-sectional and prospective evidence from the Scottish health survey. Arch Gen Psychiatry.

[CR49] Hu L-T, Bentler PM (1999). Cutoff criteria for fit indexes in covariance structure analysis: conventional criteria versus new alternatives. Struct Equ Model Multidiscip J.

[CR50] Hutchinson G, Simeon DT, Bain BC, Wyatt GE, Tucker MB, LeFranc E (2004). Social and health determinants of wellbeing and life satisfaction in Jamaica. Int J Soc Psychiatry.

[CR51] Jin-Lee Y, Young WL, Ji Y, Yang C, Soo K, Young CS, Dong CS (2011). Evaluating the PM damage cost due to urban air pollution and vehicle emissions in Seoul, Korea. J Environ Manag.

[CR52] Kahneman D, Krueger A, Schkade D, Schwarz N, Stone A (2006). Would you be happier if you were richer? A focusing illusion. Science.

[CR53] Kapteyn A, Lee J, Zamarro G (2013) ‘Does retirement induced through social security pension eligibility influence subjective well-being?’ A cross-country comparison. Working Paper WP 2013–301. Michigan Retirement Research Center University of Michigan

[CR54] Kopelman P (2007). Health risks associated with overweight and obesity. Short science review. Foresight tackling obesities: future choices. Obes Rev.

[CR55] Krupnick A, Alberini A, Cropper M, Simon N, O’Brien B, Goeree R, Heintzelman M (2002). Age, health and the willingness to pay for mortality risk reductions: a contingent valuation survey of Ontario residents. J Risk Uncertain.

[CR56] Lee BJ, Kim B, Lee K (2014). Air pollution exposure and cardiovascular disease. Toxicol Res.

[CR57] Lelieveld J, Evans SJ, Fnais M, Giannadaki D, Pozzer A (2015). The contribution of outdoor air pollution sources to premature mortality on a global scale. Nature.

[CR58] Li F, Liu Y, Lü J, Liang L, Harmer P (2015). Ambient air pollution in China poses a multifaceted health threat to outdoor physical activity. J Epidemiol Community Health.

[CR59] Lim Y-H, Kim H, Kim JH, Bae S, Park HY, Hong Y-C (2012). Air pollution and symptoms of depression in elderly adults. Environ Health Perspect.

[CR60] Luechinger S (2009). Valuing air quality using the life satisfaction approach. Econ J.

[CR61] Luechinger S (2010) Life Satisfaction and Transboundary Air Pollution. Econ Lett 1071:4–6

[CR62] Luechinger S, Raschky P (2009). Valuing flood disasters using the life satisfaction approach. J Public Econ.

[CR63] MacKerron G, Mourato S (2009). Life satisfaction and air quality in London. Ecol Econ.

[CR64] Mahmud M (2009). On the contingent valuation of mortality risk reduction in developing countries. Appl Econ.

[CR65] McConnell R, Berhane K, Yao L, Jerrett M, Lurmann F, Gilliland F, Künzli N, Gauderman J, Avol E, Thomas D, Peters J (2006). Traffic, susceptibility, and childhood asthma. Arch Environ Health.

[CR66] Mehta JA, Kubzansky LD, Coull BA, Kloog I, Koutrakis P, Sparrow D, Spiro A, Vokonas P, Schwartz J (2015). Associations between air pollution and perceived stress: the Veterans Administration Normative Aging Study. Environ Health.

[CR67] Meijer C, Wouterse B, Polder J, Koopmanscha M (2013). The effect of population aging on health expenditure growth: a critical review. Eur J Ageing.

[CR68] O’Neill MS, Loomis D, Borja-Aburto VH (2004). Ozone, area social conditions and mortality in Mexico City. Environ Res.

[CR69] Oosterlee A, Drijver M, Lebret E, Brunekreef B (1996). Chronic respiratory symptoms in children and adults living along streets with high traffic density. Occup Environ Med.

[CR70] Ostro B, Broadwin R, Green S, Feng WY, Lipsett M (2006). Fine particulate air pollution and mortality in nine California counties: results from CALFINE. Environ Health Perspect.

[CR71] Ozdamar O, Giovanis E (2016). The causal effects of income support and housing benefits on mental well-being: an application of a Bayesian network. Metroeconomica.

[CR72] Parson EA (2003). Protecting the ozone layer: science and strategy.

[CR73] Pearl J (2012). The causal foundations of structural equation modeling. R. H. Hoyle (Ed.), Handbook of structural equation modeling.

[CR74] Persinger MA (1980). The weather matrix and human behaviour.

[CR75] Pischke JF (2011) Money and happiness: evidence from the industry wage structure. Discussion Paper No. 5705, IZA

[CR76] Power MC, Kioumourtzoglou MA, Hart JE, Okereke OI, Laden F, Weisskopf MG (2015). The relation between past exposure to fine particulate air pollution and prevalent anxiety: observational cohort study. Br Med J.

[CR77] Prince M, Reischies F, Beekman ATF (1999). Development of the EURO-D scale—a European Union initiative to compare symptoms depression in 14 European centres. Br J Psychiatry.

[CR78] Rohwedder S, Willis RJ (2010). Mental retirement. J Econ Perspect.

[CR79] Shima M, Nitta Y, Adachi M (2003). Traffic-related air pollution and respiratory symptoms in children living along trunk roads in Chiba Prefecture, Japan. J Epidemiol.

[CR80] Smith VK, Huang JC (1995). Can markets value air quality? A meta-analysis of hedonic property value models. J Polit Econ.

[CR81] Smith JP, Kington R (1997). Demographic and economic correlates of health in old age. Demography.

[CR82] Stiglitz JE, Sen A, Fitoussi J-P (2009) Commission on the measurement of economic performance and social progress. http://www.stiglitz-sen-fitoussi.fr/documents/rapport_anglais.pdf

[CR83] Szyszkowicz M, Willey JB, Grafstein E, Rowe BH, Colman I (2010). Air pollution and emergency department visits for suicide attempts in Vancouver, Canada. Environ Health Insights.

[CR84] Tietenberg T, Lewis L (2009). Environmental & natural resource economics.

[CR85] Tucker LR, Lewis C (1973). A reliability coefficient for maximum likelihood factor analysis. Psychometrika 38(1):1–10

[CR86] van Praag B, Ferrer-i-Carbonell A (2004). Happiness quantified: a satisfaction calculus approach.

[CR87] Van Vliet P, Knape M, De Hartog J, Janssen N, Hassema H, Brunekreef B (1997). Motor vehicle exhaust and chronic respiratory symptoms in children living near freeways. Environ Res.

[CR88] Veenhoven R (1993). Happiness in nations, subjective appreciation of in 56 nations 1946–1992.

[CR89] Wang H, Mullahy J (2006). Willingness to pay for reducing fatal risk by improving air quality: a contingent valuation study in Chongqing, China. Sci Total Environ.

[CR90] Welsch H, Kuhling J (2009). Using happiness data for environmental valuation: issues and applications. J Econ Surv.

[CR91] WHO (2014) http://www.who.int/mediacentre/news/releases/2014/air-pollution/en/. Accessed 15 June 2017

[CR92] Wilhelm M, Ritz B (2003). Residential proximity to traffic and adverse birth outcomes in Los Angeles County, California, 1994–1996. Environ Health Perspect.

